# Strong Associations Between the Pesticide Hexachlorocyclohexane and Type 2 Diabetes in Saudi Adults

**DOI:** 10.3390/ijerph110908984

**Published:** 2014-08-29

**Authors:** Abdulaziz Al-Othman, Sobhy Yakout, Sherif H. Abd-Alrahman, Nasser M. Al-Daghri

**Affiliations:** 1 College of Applied Medical Sciences, King Saud University, Riyadh 11451, Saudi Arabia; E-Mail: amothman@ksu.edu.sa; 2 Biomarkers of Osteoporosis, Biochemistry Department, College of Science, King Saud University, Riyadh 11451, Saudi Arabia; E-Mails: sobhy.yakout@gmail.com (S.Y.); drsherif_hussein@yahoo.com (S.H.A.-A.); 3 Biomarkers Research Program, Biochemistry Department, College of Science, King Saud University, Riyadh 11451, Saudi Arabia

**Keywords:** type 2 diabetes mellitus, organochlorine pesticides, metabolic syndrome

## Abstract

Pesticide exposure has been implicated as an environmental risk factor for the development of type 2 diabetes mellitus (T2DM). The aim of this study was to investigate the association of the body burden of the pesticide hexachlorocyclohexane (HCH) with the risk of T2DM in a sample of adults from Saudi Arabia. Serum samples were obtained from 280 adult subjects. Hexachlorocyclohexane isomer residues were measured by high-resolution gas chromatography-mass spectrometry. Data on lifestyle, dietary habits, and health status were gathered. Associations between exposure and T2DM were analyzed by logistic regression. Around 49% of adults enrolled in this study were diagnosed with T2DM. Among various HCH isomers, serum concentrations of the pesticides β and γ-HCH were most strongly and consistently linked to T2DM in our studied subjects. Associations of HCH varied across five components of the metabolic syndrome. It positively and significantly associated with four out of the five components, especially elevated triglycerides, high fasting glucose, high blood pressure and HOMA-IR but negatively and significantly with HDL-cholesterol. This study in line with earlier ones about diabetes associated with HCH pesticide exposure and proposes possible hormonal pathways worthy of further investigation.

## 1. Introduction

In 2012, the International Diabetes Federation (IDF) estimated that 371 million people in the world had type 2 diabetes mellitus (T2DM) and this number will likely increase in the future [1]. In Saudi Arabia the rise in T2DM prevalence began secondary to the fast industrial development [2], and there is an increase in the T2DM trend among adult Saudis [3,4,5]. One study done on data from a large group of patients collected from 1995 to 2000, showed that 1 in 5 adult Saudis had T2DM [6]. This rising trend has been linked to both genetic and environmental factors [7].

Reported risk factors for T2DM include obesity, sedentary lifestyle, diet, family history, race/ethnicity, age, impaired fasting glucose, high blood pressure, low high-density lipoprotein cholesterol, and high triglycerides [8]. The prevalence of T2DM worldwide was 2.8% in 2000 and is estimated to reach 4.4% in 2030 [1]. Considering that only 6% of T2DM can be explained by heritable factors [9], environmental factors may have a major contribution in the increasing incidence of this disease [10].

Persistent organic pollutants (POPs) include organochlorine pesticides (OCPs) and lipophilic chemicals with long half-lives are major environmental issues because they are potentially related to various human health problems [11]. In particular, it has been recently observed that there is a relation between T2DM and OCP exposure, even at low dose. There is an increasing body of literature showing associations of OCP exposure with T2DM risk and insulin resistance [12,13,14,15].

Due to their great toxicity and environmental persistence, OCPs were banned during 1970s–1980s [16]. However, developing countries in Asia, Latin America and Africa are still producing and using some kinds of OCPs because of their effectiveness as pesticides and low cost [17]. Hexachlorocyclohexane (HCH), as one of OCPs, exists as four isomers; the most toxic form, g-HCH or γ-HCH or lindane, has been used as an insecticide for crops and also as a topical treatment for head lice and scabies.

Contaminated foods, drinking water and air ingestion are the main sources of these compounds. Technical HCH and lindane were the most widely used OCPs until the 1990s [18], while India and some developing countries have recently started producing lindane [19]. To our knowledge, the association between OCPs and T2DM has not been studied in the Middle Eastern region. Hence, this study was done to assess whether T2DM risks in Saudis are correlated with OCP exposure.

## 2. Experimental Section

### 2.1. Study Population

A total of 280 (136 T2DM, 144 non-T2DM) adult Saudis (males and females), aged 18–65 years old, were included to participate in this cross-sectional study. The subjects were selected at random from the RIYADH COHORT of the Biomarkers Research Program, King Saud University (KSU), Riyadh, Kingdom of Saudi Arabia (KSA). The Biomarker Screening in Riyadh is a database of ~10,000 Saudi subjects randomly selected from the different urban and rural Primary Health Care Centres (PHCC) in the Riyadh. Recruitment was done from individual homes which were also selected at random. Interested subjects were then requested to visit their respective PHCCs for clinical assessment and blood extraction. Ethical approval was obtained from the Ethics Committee of the College of Medicine Research Center, KSU, Riyadh, KSA, prior to commencement of research. All subjects were questioned using a questionnaire about their demographic characteristics, health performances, and medical history. Body weight and height were measured in light clothing without shoes. Blood samples were taken from the patients after at least 8 h overnight fasting. HDL and total cholesterol, fasting glucose, and triglycerides were measured by a chemical analyzer (Konelab, Espoo, Finland).

### 2.2. Measurements

The different metabolic parameters such as serum leptin, adiponectin and insulin were determined by immunoassay using a Luminex multiplex (Luminex Corporation, Austin, TX, USA), which utilizes a new fluorescent microbead technology, allowing simultaneous quantitation of several target proteins within the single serum sample of 50–100 μL. The intra-assay variation was 1.4%–7.9% and inter-assay variation of <21% for multiplex parameters assay. Minimum detectable concentration (MDC) were as follows: insulin: 50.9 pg/mL; leptin: 85.4 pg/mL; adiponectin: 145.4 pg/mL; resistin: 6.7 pg/mL and TNF-α: 0.14 pg/mL. IL-6 was determined using enzyme-linked immune-sorbent assays (ELISA) (Bioscource, Nivelles, Belgium) with an intra-assay variability of 5.5%–6.0% and inter-assay variation of 11.6%–13.8%. Fasting blood glucose and lipid profile were assessed using an auto analyzer (Konelab) and concentrations of low density lipoprotein cholesterol were calculated using Friedewald’s formula. HOMA-IR was calculated as fasting insulin (μU/mL) fasting glucose (mmol/L)/22.5. Anthropometric measurements including weight, height and waist circumference will be collected using standard and well established methods. The Body Mass Index (BMI) will be calculated by using the formula, weight (kg)/height (m^2^).

#### Pesticide Analysis

Serum α-HCH, β-HCH, γ-HCH and δ-HCH levels were determined by gas chromatography–mass spectrometry (GC/MS) as described previously [20], with minor modifications. Each sample (0.5 mL of serum) was put into a 10 mL centrifuge tube. A 2 × 5 mL aliquot of a 5:1 mixture of hexane and acetone was added, shaken for one minute on a vortex and centrifuged at 3000 rpm for 5 min. The supernatant was then collected in a new tube and transferred to a SPE cartridge previously added with l.0 g of sodium sulphate. Two different eluents were used [E1: 6 mL *n*-hexane and E2: 6 mL mixture of *n*-hexane-Cl_2_CH_2_ (5:1 *v*/*v*)]. The eluates were concentrated to 1.0 mL and filtered using a 0.45 μm PTFE syringe filter. The sample was then ready for analysis. The limit of detection (LOD) was defined as the higher of either the method blank LOD (three times standard deviation of method blanks after subtracting the average blank) or the instrument LOD (signal >3 times the signal to noise ratio) and the limit of quantification (LOQ) (signal >10 times the signal to noise ratio). LOD ranged from 1.0 to 5.0 ng∙mL^−^^1^.

The calibration curve and recovery validation study were all repeated three times. Recovery and precision were estimated by using spiked blank matrix samples analyzed in duplicate at five levels spread equally over the analytical range. The recoveries were calculated from the analytical signal as the ratio between found and expected expressed in percent. The rate of recovery for all five OCPs ranged from 85% to 103%.

### 2.3. Statistical Analysis

Statistical analyses has been done using SPSS version 11.5 (SPSS Inc., Chicago, IL USA). Descriptive statistics was done using percentage for frequencies, mean ± standard deviation for normally distributed continuous variables and median (range) for those otherwise. Pearson and multivariate regression analyses were done to determine associations of interest as well as significant predictors between the different phenotype measures to the log-transformed hormonal profiles and POCs while adjusting for BMI, gender, age, socio-economic status, *etc.* Multiple logistic regression analyses was carried to identify significant predictors for POC exposure and associated T2DM risk. Independent T-test was also done (Mann-Whitney for non-Gaussian variables) adjusting for significant covariates to determine differences in hormonal expression and POC level within groups.

## 3. Results and Discussion

Table 1 shows the general characteristics of the final study population. Out of the 280 adults enrolled in this study, 136 (49%) were diagnosed with T2DM. BMI, triglycerides and fasting plasma glucose were significantly higher in cases while HDL cholesterol was significantly higher in controls Serum samples were analyzed for three hexachlorocyclohexane isomers (α-, β-, and γ-). In Section 2.2, four isomers T2DM patients have significantly higher concentrations of the γ-hexachlorocyclohexane isomer than controls (Table 1). Geometric mean HCH concentrations ranged from 0.41 ng/mL (α-HCH) to 7.3 ng/mL (γ-HCH) in the whole sample. Serum γ-HCH had the highest concentration among the isomers in both groups. Repetitive Frequencies of detection of HCH isomers in T2DM were 29.3% (α-HCH), 44.3% (β-HCH), and 51.1% (γ-HCH).

**Table 1:** Main characteristics of the study subjects according to group.

Parameters	Control	Diabetes	*P*
*N*	144	136	
Gender (M/F)	49/95	60/76	0.08
Age (years)	37.4 ± 6.6	43.0 ± 6.8	<0.001
BMI (kg/m^2^)	29.3 ± 5.9	32.2 ± 5.3	<0.001
Waist (cm)	92.6 ± 16.4	94.2 ± 24.7	0.11
Hips (cm)	100.4 ± 27.7	106.3 ± 28.6	0.09
Sagittal Abdominal Diameter (cm)	22.4 ± 5.1	23.4 ± 7.0	0.30
Systolic BP (mmHg)	111.9 ± 1.3	122.6 ± 10.3	<0.001
Diastolic BP (mmHg)	73.9 ± 7.5	79.1 ± 7.4	<0.001
Total Cholesterol (mmol/L)	5.0 ± 1.1	5.1 ± 1.1	0.56
Glucose (mmol/L)	5.2 ± 0.79	11.1 ± 4.0	<0.001
HDL-Cholesterol (mmol/L)	2.4 ± 0.51	0.88 ± 0.32	<0.001
HbA1c (mmol/L)	5.1 ± 0.50	8.0 ± 1.6	<0.001
Triglycerides (mmol/L)	1.2 ± 0.86	2.1 ± 0.96	<0.001
Insulin (IU/mL)	15.3 ± 4.3	32.0 ± 6.5	<0.001
HOMA-IR	1.6 ± 0.54	5.2 ± 2.3	<0.001
Leptin (ng/mL)	18.3 ± 5.3	26.5 ± 7.3	<0.001
Resistin (ng/mL)	3.4 ± 0.86	4.5 ± 0.95	0.006
Alpha-HCH (ng/mL) *	0.28 ± 0.05	0.41 ± 0.06	0.15
Beta-HCH (ng/mL) *	0.62 ± 0.09	0.75 ± 0.09	0.35
Gamma-HCH (ng/mL) *	2.8 ± 0.49	7.3 ± 0.84	<0.001
Sum HCH(ng/mL)	3.8 ± 0.51	8.8 ± 0.88	<0.001

Note: * Data represented by Mean ± standard Error; *p*-value significant at *p* < 0.05.

**Figure 1:**
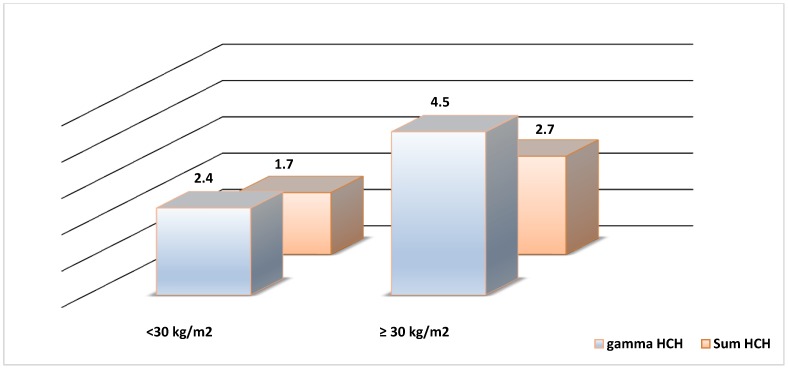
Odds ratios (OR) for Diabetes in gamma-HCH, sum HCH and two levels of Body Mass Index (BMI).

Associations of α, β, and γ-HCH with all studied parameters are presented in Table 2. HCH was significantly associated with HDL-cholesterol, glucose, triglycerides, systolic blood pressure and HOMA-IR. Adipokine levels did not significantly change upon exposure to HCH. In the unadjusted models, T2DM showed a statistically significant association with serum concentrations of all HCH isomers measured (Table 3). However, when models were further adjusted for age and BMI (model 2), and adjusted for triglycerides and cholesterol (model 3), only γ-HCH remained significantly associated with T2DM with [odds ratios 2.0 (1.1, 3.9) and 1.8 (1.1, 3.2), respectively] (Figure 1). Log-transformed variables displayed similar positive associations at all models.

This study is to our knowledge the first study in Saudi Arabia to evaluate the potential effects of pesticides in T2DM. We found that organochlorines pesticide levels determined as serum HCH were significantly correlated with T2DM in the adult Saudi population, even after adjustment for age, BMI, serum triglycerides and cholesterol. There is an increasing body of evidence that environmental exposure to POP compounds is related with an increased incidence of T2DM disease. Previous cross-sectional, longitudinal studies and a meta-analysis, with different adjustment levels, reported that serum levels of OCPs and/or PCBs were positively associated with T2DM risk [21,22,23,24]. In addition, there is a correlation between high T2DM prevalence among farmers exposed to plant pesticide [25]. With respect to HCH, our results are consistent with others who found significantly higher concentrations of HCH (especially the β-isomer) in patients with T2DM than those without [26,27,28].

The proportion of people with T2DM have increased throughout Asia, especially in the Gulf countries. Asians tend to develop diabetes at a lower BMI and at younger ages, suffer longer with complications, and die sooner than people in other regions [29]. In addition to rapid lifestyle change, a strong genetic susceptibility to T2DM, characterized by early β-cell failure and prominent central obesity, is currently regarded as a cause for the current epidemic of T2DM in Saudi Arabia.

Technical hexachlorocyclohexane and lindane (γ-HCH) were among the most extensively used OCPs produced mainly after the Second World War until the 1990s [18]. Their use has been banned or restricted in most countries for more than 30 years [30]. However, lindane remains commercially available not only in India but in other developing countries [19,31].

**Table 2:** Spearman correlation coefficients among wet-weight and lipid-standardized serum concentrations of organochlorine pesticides in controls.

Parameters	α-HCH (ng/mL)	β-HCH (ng/mL)	γ-HCH (ng/mL)	Sum HCH (ng/mL)
Age (years)	−0.009	0.01	0.13	0.14
BMI (kg/m^2^)	−0.04	0.008	0.07	0.07
Waist (cm)	0.09	−0.08	−0.01	0.04
Hips (cm)	−0.09	−0.08	−0.05	−0.05
SAD (cm)	0.04	−0.04	0.006	0.005
Systolic BP (mmHg)	0.11	0.09	0.19 **	0.23 **
Diastolic BP (mmHg)	0.04	0.09	0.13	0.16
Total Cholesterol (mmol/L)	−0.02	0.04	0.01	0.005
Glucose (mmol/L)	0.08	0.09	0.23 **	0.26 **
HDL (mmol/L)	−0.07	0.06	−0.14	−0.13 *
LDL(mmol/L	0.07	−0.01	0.07	0.08
Triglycerides (mmol/L)	0.06	0.007	0.15 **	0.16 **
Insulin (IU/mL)	−0.03	0.09	0.09	0.09
HOMA-IR	0.04	0.39 **	0.16	0.22 **
HbA1c (%)	0.06	0.05	--	--
Adiponectin (μg/mL)	−0.03	−0.06	0.05	−0.01
Leptin (ng/mL)	0.02	0.04	−0.02	−0.01
Resistin (ng/mL)	−0.02	0.02	0.12	0.12

Values presented as coefficients (R); * denotes significance at 0.05 level; ** denotes significance at 0.01 level; *p*-value significant at < 0.05.

OCP residues are still detected in water, air, soil and animals globally. These are residues from past applications and continued use by some countries [30]. Exposure to these compounds include ingestion of contaminated foods (fatty fish, animal fats and dairy products) or ingestion of contaminated water. The isomer γ-HCH is rapidly metabolized in 8 days to β-HCH, which has a half-life of about 7.6 years in humans [32,33]. In workers engaged in the production and formulation of γ-HCH, mean plasma levels ranged from 16–57 µg/L [34]. In another γ-HCH-producing facility, γ-HCH air levels of 0.004–0.15 mg/m^3^ were associated with serum levels of 5–188 µg/L in exposed workers of [35]. The high serum γ-HCH concentrations in our study in both controls (2.8 ± 0.49 μg/L) and T2DM patients (7.3 ± 0.84) indicate a recent or current exposure to γ-HCH either through diet, drinking water or polluted air. γ-HCH was also found in 23.5% of screened breast milk samples obtained from mothers in Al-Kharj, Saudi Arabia [mean concentrations of 1.061 µg/L and 23.3 µg/kg milk fat] [36]. In addition, it should be noted that circulating adiponectin, resistin and leptin were not affected after HCH exposure. Hence, OCP exposure does not seem to influence inflammatory response in mature adipocytes.

**Table 3:** Multiple logistic regression models for POP concentrations and diabetes

Parameters	Model 1	Model 2	Model 3	Model 4
α-HCH (ng/mL)	1.7 (1.0, 3.0) *	1.9 (0.92, 4.2)	1.6 (0.86, 2.9)	1.7 (0.72, 4.3)
Female	1.4 (0.687, 2.9)	1.6 (0.59, 4.5)	1.3 (0.57, 3.0)	1.5 (0.35, 6.2)
Male	2.1 (0.85, 4.9)	2.3 (0.72, 7.2)	2.1 (0.79, 5.2)	2.1 (0.60, 7.2)
β-HCH (ng/mL)	1.6 (1.0,2.7) *	1.1 (0.58, 2.2)	1.6 (0.94, 2.9)	0.96 (0.43, 2.1)
Female	1.5 (0.81, 2.8)	1.1 (0.47, 2.7)	1.3 (0.62, 2.9)	1.1 (0.31, 3.6)
Male	1.8 (0.83, 4.1)	1.0 (0.38, 3.0)	2.2 (0.90, 5.4)	1.0 (0.32, 3.3)
γ-HCH (ng/mL)	1.8 (1.2, 2.9) *	2.0 (1.1, 3.9) *	1.8 (1.1, 3.2) *	1.8 (0.86, 3.9)
Female	2.1 (1.1, 3.8) *	2.8 (1.2, 6.6) *	2.9 (1.3, 5.8) *	3.9 (1.1, 6.8) *
Male	1.4 (0.67, 3.1)	1.3 (0.49, 3.7)	1.3 (0.56, 2.9)	1.2 (0.40, 3.6)
Sum HCH (ng/mL)	2.8 (1.6, 4.4) *	2.8 (1.4, 5.4) *	3.2 (1.8, 5.5) *	2.7 (1.3, 6.0) *
Female	2.4 (1.3, 4.6) *	3.2 (1.3, 7.8) *	3.3 (1.9, 7.6) *	4.3 (2.1, 8.3) *
Male	3.2 (1.4, 7.0) *	2.6 (0.90, 7.4)	3.0 (1.2, 7.2) *	2. 1(0.72, 7.2)

Model 1 Crude Odds ratio; Model 2 Adjusted for Age and BMI; Model 3 Adjusted for Triglycerides and Cholesterol; Model 4 Adjusted for Age BMI, Triglyceride and Cholesterol; Odd ratios in bold and with asterisk * denotes *p*-value<0.05.

Poor glycemic control caused by HCH may increase risk for T2DM complications. The positive association between HCH pesticides and HOMA-IR supports this possibility. Several studies reported that PCB concentrations were associated with elevated levels of blood glucose [37,38]. HCH serum concentrations were also associated with adverse lipid profiles like high triglycerides and low HDL-cholesterol even among subject without T2DM [38]. Elevated hypertriglyceridemia is a risk factor for insulin resistance and a clinical biomarker for metabolic syndrome [39] which may have high bearing on cardiovascular health. Several studies have clearly demonstrated that some pesticides cause alterations in lipid profile, particularly triglycerides [40,41].

Consistent findings of a higher risk for T2DM in obese subjects led researchers to propose that adipose tissue plays an important role in the pathogenesis of this disease as well as cancer [42,43]. Others reported that the risk of T2DM is lower in persons with a low POPs body burden, independent of BMI. This shows that POPs deposited in adipose tissue may be promoters of T2DM [13]. This proposition is supported by the present finding of a positive trend in the OR for diabetes in non-obese subjects with increased levels of HCHs. Furthermore, it has been reported that POPs may induce obesity (obesogenic effect) [42].

The mechanisms describing the association between T2DM and organochlorines are still inexact. The most likely mechanism is one involving tissue specific up- or down regulation of gene expression, which might promote glucose intolerance. It is well known that the toxic effects of several POPs are mediated via binding with aryl hydrocarbon receptor (AhR), a ligand-activated transcription factor. It has been suggested that this receptor may promote diabetogenesis by antagonizing the functions of peroxisome proliferator-activated receptors, recently found to be linked to cellular proliferation, differentiation and apoptosis, as well as to obesity, diabetes, atherosclerosis, inflammation, cancer and ageing [44]. However, POPs’ diabetogenic induction may also be promoted through mitochondrial dysfunction and AhR-independent oxidative stress [45]. Fischer *et al.* observed that insulin release and intracellular Ca^2+^ rise from RINm5F cells promoted by PCB47 and PCB153 which have small AhR affinity [46]. In addition, Biswas *et al*. observed that 2,3,7,8-tetrachlorodibenzo-*p*-dioxin, a high-affinity AhR, triggers mitochondrial dysfunction in mouse skeletal muscle C2C12 myoblasts, independent of AhR activation [47].

Our findings have several limitations. First, the associations observed between HCH and diabetes does not establish causality. This investigation was not originally designed as a health study, so some important risk factors for T2DM were not determined at baseline (smoking, alcohol use, physical activity, and family history of T2DM). Second, as our study had a small sample size, we could not rule out that some associations were findings by chance. Stratified analyses were impossible due to a small sample size. Third, only single measurements were made for both fasting glucose level and serum HCH levels. Although participants were instructed to fast overnight before providing blood samples, it could not be objectively confirmed that they did so, and glucose can vary significantly in the non-fasting state. If some participants did not fast as instructed, measurement bias could affect our findings. However, this bias is likely to be non-differential because there is no reason to suppose that participants with a higher toxicant burden would have been preferentially more inclined not too fast.

## 4. Conclusions

In conclusion, we found that serum concentrations of OCPs were significantly associated with T2DM risk in Saudi adults who had background exposure to OCPs. Moreover, the absolute serum concentrations of OCPs were higher in T2DM than non-T2DM. We hypothesize that Saudis are more susceptible to the adverse effects of OCPs than other ethnic groups, partly explaining the current epidemic of T2DM in Saudi Arabia. Future studies should address the biological mechanisms by which OCPs may be affecting glucose homeostasis.
